# Cardiotoxicity caused by acrylamide in rats can be alleviated as a result of suppression of oxidative stress, endoplasmic reticulum stress, inflammation, and apoptosis by morin treatment

**DOI:** 10.22038/ijbms.2024.81490.17634

**Published:** 2025

**Authors:** Fatma Cakmak, Sefa Kucukler, Cihan Gur, Mustafa Ileriturk, Murat Gul, Behcet Varisli

**Affiliations:** 1 İstanbul Atlas University, Faculty of Medicine, Department of Emergency Medicine, İstanbul, Turkey; 2 Atatürk University, Faculty of Veterinary Medicine, Department of Biochemistry, Erzurum, Turkey; 3 Atatürk University, Vocational School of Health Services, Department of Medical Laboratory Techniques, Erzurum, Turkey; 4 Atatürk University, Horasan Vocational College, Department of Animal Science, Erzurum, Turkey; 5 Aksaray University, Faculty of Medicine, Department of Cardiology, Aksaray, Turkey; 6 Final Inernational University, Vocational School of Health Services, Cyprus, Turkey

**Keywords:** Apoptosis, Acrylamide, Cardiotoxicity, Endoplasmic reticulum – stress, Morin

## Abstract

**Objective(s):**

The present study investigated whether morin has a protective effect against ACR-induced cardiac toxicity.

**Materials and Methods:**

In this study, oxidative stress, inflammation, endoplasmic reticulum stress (ERS), and apoptosis markers in heart tissues were analyzed by different methods after ACR (38.27 mg/kg) and morin (50 or 100 mg/kg) oral administration for ten days to Sprague Dawley rats.

**Results:**

The data obtained showed that ACR induced lipid peroxidation by decreasing superoxide dismutase (SOD), catalase (CAT), and glutathione peroxidase (GPx) enzyme activities, glutathione (GSH) levels and nuclear factor erythroid 2-related factor 2 (Nrf-2), heme oxygenase-1 (HO-1), NAD(P)H dehydrogenase quinone 1 (NQO1), glutamate-cysteine ligase modifier subunit (GCLM), and glutamate-cysteine ligase catalytic subunit (GCLC) gene expressions. On the other hand, these markers approached the control group levels after morin treatment. Moreover, morin suppressed ACR-induced inflammatory genes. Morin down-regulated the related genes by reducing the ERS, exacerbated after ACR administration. In addition, it was observed that B-cell lymphoma-2 (Bcl-2) associated X protein (Bax), caspase-3, and apoptotic peptidase activating factor 1 (apaf-1) expressions, elevated by ACR in the heart tissue, were suppressed after morin administration. Moreover, Bcl-2 expression was triggered by morin treatment. Thus, morin suppressed ACR-induced apoptosis.

**Conclusion:**

Taken together, morin may protect against ACR-induced cardiac injury by suppressing oxidative stress, inflammation, ERS, and apoptosis.

## Introduction

Environmental pollution is a global problem that threatens many living groups, including humans, and unfortunately, increases day by day due to conscious or unconscious activities of people. Many factors that cause environmental pollution have an important role in the etiology of various diseases (1). Acrylamide (ACR), which is among these factors and extracted from hydrated acrylonitrile, is a compound with extremely high water solubility and chemical activity (1, 2). Polyacrylamide, a polymeric version of ACR, is used in cosmetics, plastics, varnishes and adhesives, paints, laboratory supplies, textiles, and paper production (1-3). In addition to being found in tobacco smoke, ACR is also of great concern because it occurs in the Maillard reaction while processing foods with high carbohydrate content, such as bread, French fries, and roasted coffee (1, 4). ACR, which can pass into the human body by swallowing, inhalation, or through the skin, is rapidly absorbed by the digestive system and spreads rapidly to many tissues such as the liver, heart, and kidney (3, 5). The FAO/WHO Expert Committee on Food Additives (JECFA) reported that the adult population was exposed to dietary ACR at 0.2-1.0 μg/kg body weight per day, and children at a higher rate than these values (6). After ACR enters the systemic circulation, it is converted to glycidamide, an epoxide metabolite, either by the CYP2E1 enzyme system in the liver or it is conjugated with glutathione (GSH) by glutathione-S-transferase (GST) to non-toxic N-Acetyl-S-(2-carbamoylethyl) cysteine (3, 5, 6). It has been reported that ACR, classified as a “Group 2A carcinogen” (7), causes damage to DNA and proteins by disrupting the redox balance, causing mitochondrial dysfunction, and triggering the inflammatory process (5).

The efficacy of antioxidant compounds against tissue damage caused by various toxic compounds, including pesticides, chemotherapeutics, and heavy metals, has been intensively studied (8-10). Among these compounds, flavonoids have been shown to protect against tissue damage by reducing oxidative stress and regulating pathways that play a role in the mechanism of toxicity in tissues such as the heart, liver, kidney, brain, testis, and lung (11-15). Morin, a natural polyphenolic antioxidant isolated from members of the Moraceae family (16), is a natural flavonoid with antioxidant, anti-inflammatory, antidiabetic, anticarcinogenic, neuroprotective, and antiproliferative effects (17). One of the most important advantages of morin is that it has a weak cytotoxic effect even at high doses (18). Previous studies have reported that morin has protective properties against various toxic substances in different tissues (19-21).

The present study investigated the effects of morin treatment against ACR-induced cardiotoxicity. For this purpose, oxidative stress markers malondialdehyde (MDA), GSH, superoxide dismutase (SOD), catalase (CAT), and glutathione peroxidase (GPx) were analyzed in rat heart tissues by biochemical methods, and nuclear factor erythroid 2-related factor 2 (Nrf-2), heme oxygenase-1 (HO-1), NAD(P)H dehydrogenase quinone 1 (NQO1), glutamate-cysteine ligase modifier subunit (GCLM), and glutamate-cysteine ligase catalytic subunit (GCLC) were analyzed by RT-PCR method. Additionally, B-cell lymphoma-2 (Bcl-2) associated X protein (Bax), Bcl-2 caspase-3, and apoptotic peptidase activating factor 1 (apaf-1) were analyzed by RT-PCR and/or western blot method to determine the apoptosis status. Relative mRNA transcript levels of activating transcription factor-6 (ATF-6), double-stranded RNA-activated protein kinase (PKR)-like ER kinase (PERK), inositol-requiring enzyme-1 (IRE1), C/EBP homologous protein (CHOP) and glucose-regulated protein 78 (GRP-78) genes that play a role in ER stress and nuclear factor kappa-B (NF-κB), tumor necrosis factor-alpha (TNF-α), interleukin-1 beta (IL-1β), cyclooxygenase-2 (COX-2) and inducible nitric oxide synthase (iNOS) genes that play a role in inflammation were analyzed.

## Materials and Methods

### Chemicals

All chemicals, including acrylamide (CAS Number 79-06-1) and morin hydrate (CAS No: 654055-01-3), were obtained from Sigma Chemical Company (St. Louis, MO, USA).

### Supply and care of experimental animals

Male Sprague Dawley rats, 2 to 2.5 months old and weighing 230-270 g, were used in the study. Animals were obtained from the Atatürk University Experimental Research and Application Center (Erzurum, Turkey), and all applications on animals were performed in this center. The housing environment of the rats had 24 ±1 ^°^C, 45±5% humidity, and a 12-hour light/dark cycle. Standard pellet feed and tap water (*ad libitum*) were used in their feeding. Ethics committee approval for the study was obtained from the Atatürk University Experimental Animals Local Ethics Committee (Approval No: 2022/8/158).

### Study groups

In a recent study, rats were randomly divided into five groups, each including seven animals. ACR administration was made with reference to the study of Uthra *et al*. (22). According to this study, 1/3 of the LD50 dose of ACR was given. The doses previously used by Çelik *et al*. (23) were used for morin administration. The groups were designed as follows;

1. Control Group: The rats were given saline orally for ten days.

2. Morin Group: Rats were given orally 100 mg/kg morin hydrate for ten days.

3. ACR Group: 38.27 mg/kg ACR was administered orally to rats for ten days.

4. ACR-Morin-50 Group: The rats were given 38.27 mg/kg of ACR orally for ten days, and then morin hydrate was given orally at 50 mg/kg 30 min later.

5. ACR-Morin-100 Group: The rats were given 38.27 mg/kg of ACR orally for ten days, and then morin hydrate was given orally at 100 mg/kg 30 min later.

On the 11th day of the study (24 hr after the last application), the animals were anesthetized with 200 ppm sevoflurane, decapitated, and their heart tissues removed. Afterward, heart tissues were analyzed using biochemical, molecular, and western blot techniques.

### Analysis of lipid peroxidation and enzymatic and non-enzymatic antioxidant markers in heart tissues

Heart tissues from animals were ground in liquid nitrogen with the aid of Tissue Lyser II (Qiagen, Netherlands). Then, they were diluted with 1.15% potassium chloride and homogenized in the same device. Homogenates were centrifuged at +4 ^°^C, 3500 rpm for 15 min to measure MDA levels, which are indicators of lipid peroxidation, and SOD and CAT activities, which are enzymatic antioxidants. In addition, for the analysis of glutathione peroxidase, another enzymatic antioxidant, and GSH, an antioxidant tripeptide, homogenates were centrifuged at +4 ^°^C, 10,000 rpm for 20 min. MDA levels were analyzed using the method of Placer *et al*. (1966)(24) based on the reaction of MDA with thiobarbituric acid (TBA) in the obtained supernatants. In the measurement of SOD activity, the method of Sun *et al*. (1988)(25), based on the inhibition of nitroblue tetrazolium (NBT) reduction of enzymatically produced superoxide radicals in the reaction medium by SOD in the medium was used. Aebi’s (1984)(26) method, based on the reduction in absorbance at 240 nm with the degradation of H_2_O_2_, was used to measure catalase activity. The methods of Lawrence and Burk (1976)(27) and Sedlak and Lindsay (1968)(28) were used for GPx and GSH analysis, respectively. The total protein content of heart tissues was determined by the method developed by Lowry *et al*. (1951)(29).

### RT-PCR analyzes in heart tissues

Previously ground heart tissues were used for RT-PCR analyses. Total RNA isolation was performed from powdered tissues using QIAzol Lysis Reagent (Qiagen, Cat: 79306, Germany) according to the manufacturer’s instructions. After isolation, the total RNA concentrations of the samples were measured in a NanoDrop device (Epoch Microplate Spectrophotometer, USA). According to the results obtained, total RNA concentrations were equalized at 1000 ng/µl, and then RNAs were converted into cDNAs using the High-Capacity cDNA Reverse Transcription Kit (Applied Biosystems™ Cat: 4368814, USA). In the next step, the reaction was started after the mixture was prepared in the ROTOR-GENE Q (Qiagen, Germany) device with cDNAs, the primers whose sequences are given in Table 1, and iTaq Universal SYBR Green Supermix (BIO-RAD) according to the manufacturer’s instructions. After the reaction process was completed, the genes were normalized to β-actin using the 2^-∆∆CT^ method of Livak and Schmittgen (30).

### Western blot analyses of heart tissue

For Western blot analysis in heart tissues, firstly, total proteins were isolated from tissues ground in liquid nitrogen with the help of RIPA lysis buffer (Santa Cruz Biotechnology), and their concentrations were determined with PierceTM BCA Protein Assay Kit (Rockford, IL, USA). Then, the samples were mixed with Laemmli buffer and subjected to sodium dodecyl sulfate-polyacrylamide gel electrophoresis (SDS-PAGE). Proteins separated in the gel were transferred to polyvinylidene fluoride (PVDF) membranes by blotting and blocking with 5% bovine serum albumin (BSA). Next, the membranes were incubated overnight with Bax, caspase-3, and β-actin monoclonal antibodies. After incubation, monoclonal antibodies were removed, and membranes were washed with PBST. Washed membranes were incubated with anti-mouse IgG secondary antibody (1:2000 dilution) for two hours. At the end of the period, they were washed again with PBST and visualized on the Biorad Gel Doc XR+ Imaging System (Bio-Rad, Hercules, USA) in the presence of ECL Substrate (Bio-Rad, Hercules, USA). The intensities of the bands were calculated in the ImageLab program (Bio-Rad, Hercules, USA).

### Statistical analysis

Statistical analysis of the data was performed using one-way analysis of variance (one-way ANOVA) and Tukey’s multiple comparison test in IBM SPSS (version 20.0; IBM Co, North Castle, NY, USA). The results were given as mean ± standard deviation (SD). *P*<0.05 was considered statistically significant.

## Results

### Effects of morin on acrylamide-induced lipid peroxidation in heart tissue

After ACR and morin treatments, MDA levels in the tissue were analyzed to determine lipid peroxidation in the heart tissues of rats. The results showed that ACR administration increased MDA levels by causing lipid peroxidation in heart tissue (According to the control: %80) (Table 2). Morin treatment attenuated ACR-induced lipid peroxidation and thus decreased MDA levels (ACR vs ACR-Morin-50: %24, ACR vs ACR-Morin-100: %36) (*P*<0.001). When a comparison was made between the doses of morin, it was determined that 100 mg/kg was more effective than the low dose (*P*<0.001, Table 2).

### Effects of morin on enzymatic antioxidants and GSH inhibited by acrylamide in heart tissue

The results are presented in Table 2. First of all, it should be noted that ACR inhibits the activities of these antioxidant enzymes in heart tissue and depletes GSH stores (According to the control; GSH: %54, SOD: %57, CAT: %42, GPx: %61). On the other hand, it is seen that there is an increase in the activities of SOD, CAT and GPx enzymes compared to the ACR group with morin treatment (ACR vs ACR-Morin-50; GSH: %14, SOD: %23, CAT: %22, GPx: %59) (ACR vs ACR-Morin-100; GSH: %23, SOD: %35, CAT: %32, GPx: %66). It is also noteworthy that GSH stores are also renewed after morin treatment. When morin doses were compared on these factors, it was found that a high dose was more effective than a low dose on only GSH (*P*<0.01) and CAT (*P*<0.05) markers.

### Effects of morin on antioxidant genes down-regulated by acrylamide in heart tissue

**Figure 1:**
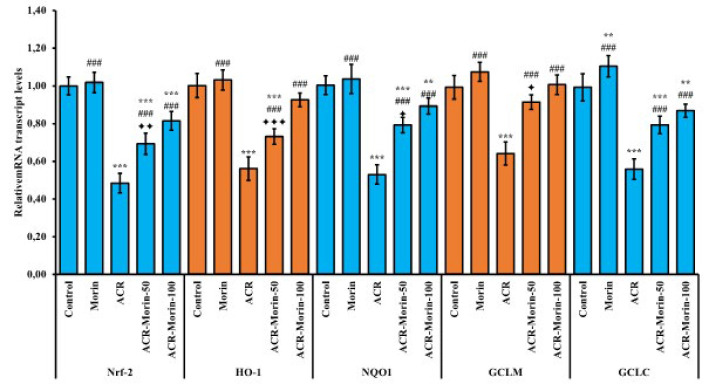
Effects of acrylamide and morin administrations on relative mRNA transcript levels of Nrf-2, HO-1, NQO1, GCLM, and GCLC genes in heart tissue of rats

After administration of ACR and morin to rats, mRNA transcript levels of Nrf-2, HO-1, NQO1, GCLM and GCLC genes were examined in heart tissues to show oxidative stress at the gene level, and the results are summarized in Figure 1. ACR appears to suppress mRNA transcript levels of all five genes in heart tissue (According to the control; Nrf-2: %52, HO-1: %44, NQO1: %47, GCLM: %35, GCLC: %44). On the other hand, morin administration up-regulated these antioxidant genes in heart tissue compared to the ACR group (ACR vs ACR-Morin-50; Nrf-2: %43, HO-1: %30, NQO1: %50, GCLM: %43, GCLC: %42) (ACR vs ACR-Morin-100; Nrf-2: %68, HO-1: %65, NQO1: %68, GCLM: %57, GCLC: %55) (*P*<0.001 for all). When comparing the doses, mRNA transcript levels of all genes except GCLC were expressed more at high doses than at low doses (Nrf-2; *P*<0.01, HO-1; *P*<0.001, NQO1 and GCLM; *P*<0.05).

### Effects of morin on acrylamide-induced inflammatory genes in heart tissue

**Figure 2:**
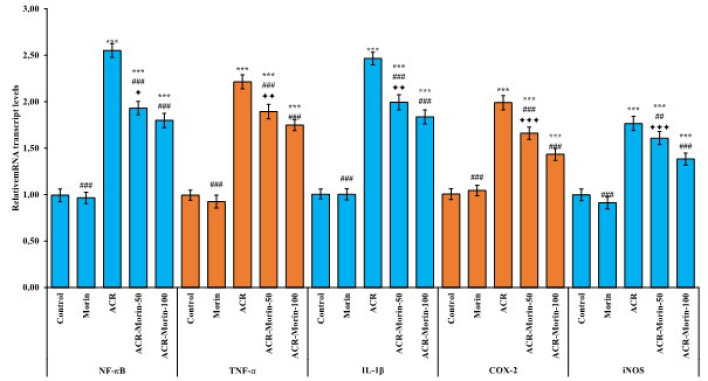
Effects of acrylamide and morin administrations on relative mRNA transcript levels of NF-κB, TNF-α, IL-1β, iNOS, and COX-2 genes in heart tissue of rats

In order to evaluate the inflammatory state in heart tissue after ACR and morin treatments, mRNA transcript levels of NF-κB, TNF-α, IL-1β, COX-2, and iNOS genes were analyzed by RT-PCR method. Obtained results are given in Figure 2. According to the results, it was determined that ACR up-regulated NF-κB, TNF-α, IL-1β, COX-2, and iNOS genes in heart tissue (According to the control; NF-κB: 2.55-fold, TNF-α: 2.22-fold, IL-1β: 2.47-fold, COX-2: 1.99-fold, iNOS: 1.77-fold). On the other hand, morin decreased the mRNA transcript levels of these genes in a dose-dependent manner (ACR vs ACR-Morin-50; NF-κB: %24, TNF-α: %15, IL-1β: %19, COX-2: %17, iNOS: %9)(ACR vs ACR-Morin-100; NF-κB: %30, TNF-α: %21, IL-1β: %25, COX-2: %28, iNOS: %22) (NF-κB; *P*<0.05, TNF-α and IL-1β; *P*<0.01, COX-2 and iNOS; *P*<0.001).

### Effects of morin on acrylamide-induced endoplasmic reticulum stress in heart tissue

**Figure 3:**
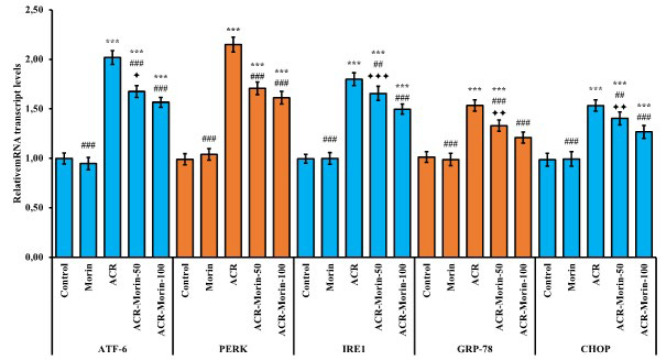
Effects of acrylamide and morin administrations on relative mRNA transcript levels of ATF-6, PERK, IRE1, GRP-78, and CHOP genes in heart tissue of rats

According to the results presented in Figure 3, ACR caused endoplasmic reticulum stress (ERS) in heart tissue and up-regulated the expressions of ATF-6, PERK, IRE1, CHOP and GRP-78 (According to the control; ATF-6: 2.02-fold, PERK: 2.15-fold, IRE1: 1.80-fold, CHOP: 1.53-fold, GRP-78: 1.53-fold). However, morin administration suppressed the expression of these genes (ACR vs ACR-Morin-50; ATF-6: %17, PERK: %21, IRE1: %8, CHOP: %8, GRP-78: %13)(ACR vs ACR-Morin-100; ATF-6: %22, PERK: %25, IRE1: %17, CHOP: %17, GRP-78: %21). While there was no difference between the low dose and high dose of morin on the mRNA transcript levels of PERK, the expressions of other genes were decreased depending on the dose (IRE1; *P*<0.001, GRP-78 and CHOP; *P*<0.01, ATF-6; *P*<0.05).

### Effects of morin on acrylamide-induced apoptosis in heart tissue

**Figure 4:**
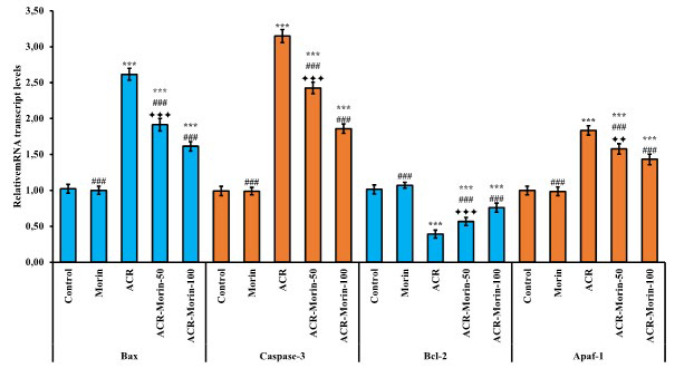
Effects of acrylamide and morin administrations on relative mRNA transcript levels of Bax, caspase-3, bcl-2, and apaf-1 genes in heart tissue of rats

To evaluate the anti-apoptotic effects of morin against ACR in heart tissue, mRNA transcript levels of Bax, Bcl-2 caspase-3, and apaf-1 genes in tissue were analyzed by RT-PCR method, and relative protein levels of Bax and caspase-3 by western blot method. RT-PCR results showed that ACR triggered Bax, caspase-3 and apaf-1 expressions (According to the control; Bax: 2.62-fold, caspase-3: 3.15-fold, apaf-1: 1.83-fold) and suppressed bcl-2 expression (According to the control; %61). However, morin down-regulated Bax, caspase-3 and apaf-1 genes (ACR vs ACR-Morin-50; Bax: %27, caspase-3: %23, apaf-1: %14) (ACR vs ACR-Morin-100; Bax: %38, caspase-3: %41, apaf-1: %22), while up-regulating bcl-2 expression by counteracting ACR (ACR vs ACR-Morin-50; Bcl-2: %45)(ACR vs ACR-Morin-100; Bcl-2: %94). Moreover, the data shows that high-dose morin is more effective than low-dose (Bax, Bcl-2, and caspase-3; *P*<0.001, apaf-1; *P*<0.01). RT-PCR results are summarized in Figure 4.

**Figure 5:**
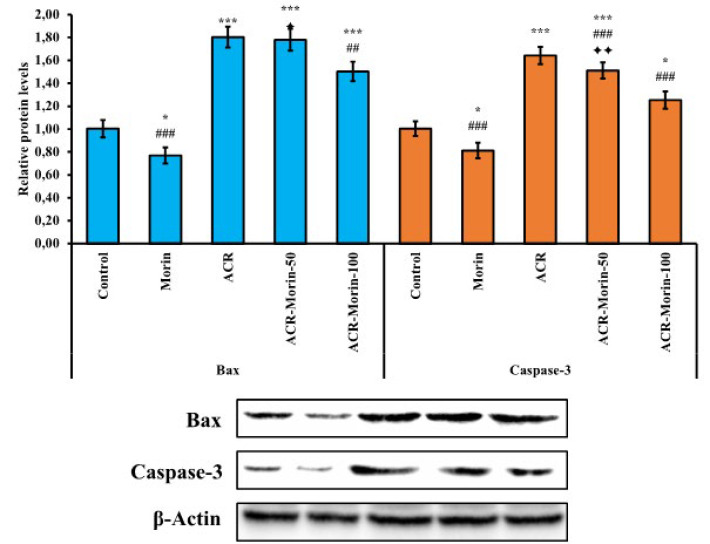
Effects of acrylamide and morin administrations on relative protein levels of Bax and caspase-3 in heart tissue of rats

Western blot results also showed that ACR increased Bax and caspase-3 protein levels, similar to mRNA transcript levels (According to the control; Bax: 1.80-fold, caspase-3: 1.64-fold). However, morin treatment appeared to reduce the levels of these proteins (ACR vs ACR-Morin-100; Bax: %17, caspase-3: %24). However, it was determined that a low dose was not effective on Bax protein levels. However, there was a dose-dependent decrease in Caspase-3 expression (*P*<0.01). Western blot results are summarized in Figure 5.

## Discussion

ACR, an environmental polluting agent, is released into the environment from various sources, especially heat-treated foods. This causes living things to be exposed to high amounts of ACR daily. ACR causes toxicity in various tissues. According to previous studies, oxidative stress is one of the important factors in ACR toxicity. For example, researchers (31) reported that ACR depleted GSH stores in the liver tissues of rats, inhibited SOD activity, suppressed Nrf-2, HO-1, NQO1, GCLM and GCLC genes, and caused lipid peroxidation. It is well known that oxidative stress triggers ERS, inflammation, and apoptosis (32-34). Therefore, suppressing oxidative stress in ACR toxicity can inhibit ERS, inflammation, and apoptosis, which have important roles in tissue damage. For this purpose, in the presented study, the protective effects of morin, an antioxidant phytochemical, against cardiotoxicity caused by ACR were investigated and positive results were obtained.

Excess cellular ROS production disrupts redox balance and decreases antioxidant enzyme activities, resulting in cytotoxicity and genotoxicity (35). This situation also forms the basis of cardiotoxicity (36, 37). Measurement of MDA levels, which is an indicator of lipid peroxidation, GSH levels, which is an antioxidant tripeptide, and the activities of antioxidant enzymes SOD, CAT, and GPx are frequently used to determine oxidative stress status (38-40). Compounds with strong antioxidant properties against oxidative stress are of great interest (41-43). One of these compounds is morin. In a previous study, it was reported that morin induces SOD, CAT, GPx, and GSH levels in cardiac tissue, which diethyl phthalate and Bisphenol-S decrease and that the MDA levels increased by these compounds decreased after morin treatment (44). In the present study, it was determined that ACR caused the depletion of stores of this tripeptide, possibly due to its conjugation with GSH and decreased SOD, CAT, and GPx activities. Moreover, suppression of the antioxidant defense system caused lipid peroxidation in the heart tissue and increased MDA levels. On the other hand, after rats were treated with morin, oxidative stress was reduced, probably due to the ROS scavenging property of morin. Morin increased SOD, CAT, and GPx activities and replenished GSH stores in cardiac tissue. In addition, morin’s strengthening of the antioxidant defense system alleviated lipid peroxidation and reduced MDA levels. Thus, morin protects against oxidative damage in cardiac tissue.

The Nrf-2-Keap1 pathway controls the expression of enzymes involved in synthesizing molecules involved in detoxification. These enzymes protect against cellular damage (11). These include the HO-1, NQO1, GCLM and GCLC genes. HO-1 is the enzyme that catalyzes the degradation of hemin and exhibits potent antioxidant properties against various stress stimuli (45). NQO1 is a cytosolic flavoprotein that catalyzes the reduction of quinones to hydroquinones. Meanwhile, it uses NADH as an electron donor and increases intracellular NAD^+^ levels. NQO1 is known to play a role in various biological activities, such as anti-inflammatory and anti-apoptotic, as well as scavenging superoxide radicals (46). GCLC and GCLM, the downstream target genes of Nrf2 signaling, regulate the intracellular ratio of GSH and GSSG. In this process, GCLC binds glutamate and cysteine, while GCLM regulates the binding of GCLC to its substrates (47). Several studies have reported that Nrf-2 inhibits cardiac remodeling and dysfunction by suppressing oxidative stress in cardiac tissue (45, 48). In the presented study, after ACR administration, it was observed that HO-1, NQO1, GCLM, and GCLC mRNA transcript levels were suppressed along with Nrf2 expression in heart tissue. In a previous study, gastric mucosal injury was induced with ketoprofen, and morin was administered as treatment. The findings have shown that morin reduces oxidative stress and protects gastric tissue by increasing Nrf-2 and HO-1 levels (49). Our study determined that Nrf-2, HO-1, NQO1, GCLM, and GCLC genes, which ACR suppresses in cardiac tissue, were up-regulated after morin treatment. This may contribute to alleviating ACR-induced oxidative stress in cardiac tissue.

Inflammation-related signaling pathways play an important role in cardiotoxicity. NF-κB, a transcription factor, contributes to the inflammatory pathway by triggering the expression of pro-inflammatory cytokines (50). Agents with various toxic effects cause the release of TNF-α, IL-1β, and iNOS together with the activation of NF-κB and thus induce cardiotoxicity by causing inflammation (51, 52). NF-κB also increases the expression of inflammatory mediators such as iNOS, which leads to NO production and activation, and COX-2, responsible for metabolizing arachidonic acids to prostaglandins (53, 54). Studies investigating the effects of ACR on the NF-κB pathway in the heart tissue are limited. Our study determined ACR up-regulated TNF-α, IL-1β, COX-2, iNOS expressions, and NF-κB expression in cardiac tissue. There is a close relationship between inflammation and oxidative stress (9, 55, 56). For this reason, morin treatment was applied against ACR with the thought that antioxidant compounds could alleviate the inflammatory process, and the results obtained suppressed the expressions of NF-κB, TNF-α, IL-1β, COX-2 and iNOS in the heart tissue of morin. In a previous study, morin treatment was applied against hepatic and cardiac injury caused by aflatoxin B1. According to the data obtained by the researchers, morin alleviated oxidative stress in both tissues and protected against inflammation by suppressing pro-inflammatory cytokines (57).

Apoptosis is cell death that occurs autonomously under the control of certain genes (58). Caspase-3 is a key protease involved in apoptosis (59, 60). Bcl-2 family proteins, whose main domain is mitochondria, are known as the main regulators of the apoptotic pathway (58). While the Bax protein activates apoptosis, the Bcl-2 protein stops the apoptotic pathway (61). An increase in the Bax/Bcl-2 ratio activates caspase-9, which in turn activates caspase-3. ACR has reportedly activated caspase-3 by increasing the Bax/Bcl-2 ratio (62, 63). Another effector of Caspase-9 is apaf-1. Cytochrome-c released from mitochondria to the cytosol forms a complex with Apaf-1, dATP, and caspase 9, and thus, caspase-9 is activated, stimulating caspase-3 and triggering the apoptotic process (64). In the present study, ACR triggered apoptotic genes in heart tissue and suppressed the expression of anti-apoptotic Bcl-2. However, morin treatment protected against ACR-induced apoptosis by bringing these genes closer to normal levels. Similarly, in a previous study, morin treatment protected against apoptosis by suppressing Caspase-3, Bax, and cytochrome c and triggering Bcl-2 and Bcl-XL in neuronal cells (65).

Apoptosis of cardiomyocytes may be associated with ERS (66). ERS occurs due to the accumulation of unfolded or misfolded proteins in the ER lumen, and oxidative stress is known to be a powerful trigger of ERS (34). Three main ERS sensors (PERK, IRE1, and ATF-6) are activated in response to ERS, and the caspase cascade is triggered. ATF-6 can also trigger CHOP, a specific pro-apoptosis protein of ERS, causing Bcl-2 to be down-regulated (67). In addition, GRP-78, an important signaling protein of ERS, is a chaperone that plays a role in the unfolded protein response (UPR) (34, 58, 68). Several studies have reported that ERS induces apoptosis in heart tissue and that relieving ERS significantly reduces cardiotoxicity (66, 67). It has been reported that ACR also causes ERS and triggers the expressions of ATF-6, PERK, IRE1, CHOP, and GRP-78; on the other hand, these markers are significantly suppressed after Rosmarinic acid treatment, which is a polyphenol compound (58). The current study determined that ACR causes ERS in heart tissue and up-regulates mRNA transcript levels of ATF-6, PERK, IRE1, CHOP, and GRP-78 genes. On the other hand, morin treatment inhibited the expression of these markers by suppressing ERS. Similarly, a previous study reported that methotrexate-induced ATF6, IRE1, PERK, and GRP78 genes were down-regulated after morin treatment (55).

**Table 1:** Sequences of primers used in RT-PCR analyses

Gene	Sequences (5’-3’)	Length (bp)	Accession No
Nrf2	F: TTTGTAGATGACCATGAGTCGC R: TCCTGCCAAACTTGCTCCAT	161	NM_031789.2
HO-1	F: ATGTCCCAGGATTTGTCCGA R: ATGGTACAAGGAGGCCATCA	144	NM_012580.2
NQO1	F: CTGGCCAATTCAGAGTGGCA R: GATCTGGTTGTCGGCTGGAA	304	NM_017000.3
GCLM	F: ACCAGTGGGCACAGGTAAAA R: CCACTCCTGGGCTTCAATGT	177	NM_017305.2
GCLC	F: TCCACTGTCCAAGGTTGACG R: GTGTCCACGTCGACTTCCAT	270	NM_012815.2
NF-B	F: AGTCCCGCCCCTTCTAAAAC R: CAATGGCCTCTGTGTAGCCC	106	NM_001276711.1
IL-1	F: ATGGCAACTGTCCCTGAACT R: AGTGACACTGCCTTCCTGAA	197	NM_031512.2
TNF-	F: CTCGAGTGACAAGCCCGTAG R: ATCTGCTGGTACCACCAGTT	139	NM_012675.3
iNOS	F: AGATCAATGCAGCTGTGCTC R: GGCTCGATCTGGTAGTAGTAGA	235	NM_012611.3
COX-2	F: AGGTTCTTCTGAGGAGAGAG R: CTCCACCGATGACCTGATAT	240	NM_017232.3
ATF-6	F: TCAACTCAGCACGTTCCTGA R: GACCAGTGACAGGCTTCTCT	130	NM_001107196.1
PERK	F: GATGCCGAGAATCATGGGAA R: AGATTCGAGAAGGGACTCCA	198	NM_031599.2
IRE1	F: GCAGTTCCAGTACATTGCCATTG R: CAGGTCTCTGTGAACAATGTTGA	163	NM_001191926.1
GRP78	F: CATGCAGTTGTGACTGTACCAG R: CTCTTATCCAGGCCATATGCAA	143	NM_013083.2
CHOP	F: GAAGCCTGGTATGAGGATCT R: GAACTCTGACTGGAATCTGG	209	NM_001109986.1
Bax	F: TTTCATCCAGGATCGAGCAG R: AATCATCCTCTGCAGCTCCA	154	NM_017059.2
Bcl-2	F: GACTTTGCAGAGATGTCCAG R: TCAGGTACTCAGTCATCCAC	214	NM_016993.2
Apaf-1	F: ACCTGAGGTGTCAGGACC R: CCGTCGAGCATGAGCCAA	192	NM_023979.2
Caspase-3	F: ACTGGAATGTCAGCTCGCAA R: GCAGTAGTCGCCTCTGAAGA	270	NM_012922.2
-Actin	F: CAGCCTTCCTTCTTGGGTATG R: AGCTCAGTAACAGTCCGCCT	360	NM_031144.3

**Table 2:** Effects of acrylamide and morin administrations on oxidative stress in heart tissue

Parameters	Control	Morin	ACR	ACR-Morin-50	ACR-Morin-100
MDA (nmol/g tissue)	63.75±2.50	60.92.02±2.53^###^	114.93±2.97^***^	87.20±2.08^***/###/^^✦✦✦^	73.27±2.26^***/###^
GSH (nmol/g tissue)	5,07±0,10	5,18±0,11^###^	2,35±0,07^***^	2,69±0,10^***/###/^^✦✦^	2,89±0,11^***/###^
SOD (U/g protein)	15,95±0,73	17,03±0,68^*^	6,85±0,42^***^	8,41±0,46^***/###^	9,27±0,56^***/###^
CAT (catal/g protein)	29,48±0,92	29,13±0,96^###^	17,00±0,68^***^	20,72±0,99^***/###/^^✦^	22,38±1,00^***/###^
GPx (U/g protein)	24,83±0,92	24,89±0,86^###^	9,59±0,59^***^	15,25±0,75^***/###^	15,97±0,75^***/###^

*, **, and *** are *P<*0.05, *P<*0.01, and *P<*0.001, respectively, and indicate the difference between the control and other groups. #, ##, ### are *P<*0.05, *P<*0.01, and *P<*0.001, respectively, and indicate the difference between ACR and other groups. ✦, ✦✦, and ✦✦✦ are *P<*0.05, *P<*0.01, and *P<*0.001, respectively, and indicate the difference between acrylamide (ACR)+morin 50 and ACR+morin 100

*, **, and *** are *P<*0.05, *P<*0.01, and *P<*0.001, respectively, and indicate the difference between the control and other groups. #, ##, and ### are *P<*0.05, *P<*0.01, and *P<*0.001, respectively, and indicate the difference between ACR and other groups. ✦, ✦✦, and ✦✦✦ are *P<*0.05, *P<*0.01, and *P<*0.001, respectively, and indicate the difference between acrylamide (ACR)+morin 50 and ACR+morin 100

*, **, and *** are *P<*0.05, *P<*0.01, and *P<*0.001, respectively, and indicate the difference between the control and other groups. #, ##, and ### are *P<*0.05, *P<*0.01, and *P<*0.001, respectively, and indicate the difference between ACR and other groups. ✦, ✦✦, and ✦✦✦ are *P<*0.05, *P<*0.01, and *P<*0.001, respectively, and indicate the difference between acrylamide (ACR)+morin 50 and ACR+morin 100

*, **, and *** are *P<*0.05, *P<*0.01, and *P<*0.001, respectively, and indicate the difference between the control and other groups. #, ##, and ### are *P<*0.05, *P<*0.01, and *P<*0.001, respectively, and indicate the difference between ACR and other groups.✦, ✦✦, and ✦✦✦ are *P<*0.05, *P<*0.01, and *P<*0.001, respectively, and indicate the difference between acrylamide (ACR)+morin 50 and ACR+morin 100

*, **, and *** are *P<*0.05, *P<*0.01, and *P<*0.001, respectively, and indicate the difference between the control and other groups. #, ##, and ### are *P<*0.05, *P<*0.01, and *P<*0.001, respectively, and indicate the difference between ACR and other groups. ✦, ✦✦, and ✦✦✦ are *P<*0.05, *P<*0.01, and *P<*0.001, respectively, and indicate the difference between acrylamide (ACR)+morin 50 and ACR+morin 100

*, **, and *** are *P<*0.05, *P<*0.01, and *P<*0.001, respectively, and indicate the difference between the control and other groups. #, ##, and ### are *P<*0.05, *P<*0.01, and *P<*0.001, respectively, and indicate the difference between ACR and other groups. ✦, ✦✦, and ✦✦✦ are *P<*0.05, *P<*0.01, and *P<*0.001, respectively, and indicate the difference between acrylamide (ACR)+morin 50 and ACR+morin 100

## Conclusion

When a general evaluation was made, it was determined that ACR suppressed antioxidant genes in the heart tissues of rats, decreased enzymatic and non-enzymatic antioxidants, and caused oxidative stress. Thus, ACR probably triggered ERS, inflammation, and apoptosis by causing chain reactions. On the other hand, ERS, inflammation, and apoptosis were suppressed due to decreased oxidative stress with morin administration. Thus, morin may protect against cardiotoxicity induced by the ACR.

## Data Availability

Not applicable.

## Declaration

We have not used any AI tools or technologies to prepare this manuscript.
